# Engineering Host Microbiome for Crop Improvement and Sustainable Agriculture

**DOI:** 10.3389/fmicb.2021.635917

**Published:** 2021-05-28

**Authors:** Sanjana Kaul, Malvi Choudhary, Suruchi Gupta, Manoj K. Dhar

**Affiliations:** School of Biotechnology, University of Jammu, Jammu, India

**Keywords:** biocontrol agents, biogeochemical processes, microbiome, microbiome engineering, omics tools, plant efficacy, mutualism

## Abstract

Dynamic consortium of microbial communities (bacteria, fungi, protists, viruses, and nematodes) colonizing multiple tissue types and coevolving conclusively with the host plant is designated as a plant microbiome. The interplay between plant and its microbial mutualists supports several agronomic functions, establishing its crucial role in plant beneficial activities. Deeper functional and mechanistic understanding of plant-microbial ecosystems will render many “ecosystem services” by emulating symbiotic interactions between plants, soil, and microbes for enhanced productivity and sustainability. Therefore, microbiome engineering represents an emerging biotechnological tool to directly add, remove, or modify properties of microbial communities for higher specificity and efficacy. The main goal of microbiome engineering is enhancement of plant functions such as biotic/abiotic stresses, plant fitness and productivities, etc. Various ecological-, biochemical-, and molecular-based approaches have come up as a new paradigm for disentangling many microbiome-based agromanagement hurdles. Furthermore, multidisciplinary approaches provide a predictive framework in achieving a reliable and sustainably engineered plant-microbiome for stress physiology, nutrient recycling, and high-yielding disease-resistant genotypes.

## Introduction

Scientific research advances over the eons of time have propelled microbial coevolution and diversification as important forces in sculpturing and carving every accessible part of nature (Saleem et al., 2017). The taxonomically diverse microbial communities interacting with different components of ecosystem are acknowledged to be a major trait in terrestrialization of plants. Therefore, this multiorganismal assemblage and its synergistic relationships with the host shape the “holobiont” framework (Cooke et al., 2019). The holistic and interactive colonization of plants by ecologically diverse microbial communities is designated as plant microbiome (Foo et al., 2017).

The microbiota can exist persistently in, on, and around different tissues during plant life cycle (Nelson, 2018). Bacteria, fungi, protozoa, archaea, and viruses comprise diverse microbiota teaming with the plant. The study of multitrophic interactions between the two has greatly elaborated ecoevolutionary and functional understanding of host-microbe interactions. Rhizosphere, endosphere, and phyllosphere are the major microecosystems where bidirectional chemical dialog directly contributes to plant development, physiology, and systemic defenses and indirectly produce root exudates and other metabolites acting as nutrient sources and signals for modulating microbial composition (Mueller and Sachs, 2015). Host genotypic traits, developmental stage, soil properties, and environmental conditions harmonize the structural and functional dynamics of microbiome (Rossmann et al., 2017). This symbiotic interactome confers many adaptive advantages to plant growth and development viz. nutrient acquisition, stress resilience, modulation of hormone levels, disease resistance, enable toxin production, and increased root exudation (Singh et al., 2020).

Opening up of new high-throughput community analyses methods, next-generation sequencing techniques and meta-“omics” tools have greatly unraveled the multitrophic interactions present in the black box of plant microbiome (Ahmad et al., 2019). With the advent of these high-throughput technologies, plant beneficial microbes can be manipulated. In this scenario, microbiome engineering may be an alternative way to understand, manipulate, and develop corresponding technologies for developing microbial communities crucial to plant health and productivity (Prasad et al., 2018). The present review fosters various practical ways by which plant-microbial mutualism can be manipulated to enhance plant performance and agricultural productivity.

## Interactive Dialog Within Plant Microbiome

**FIGURE 1:**
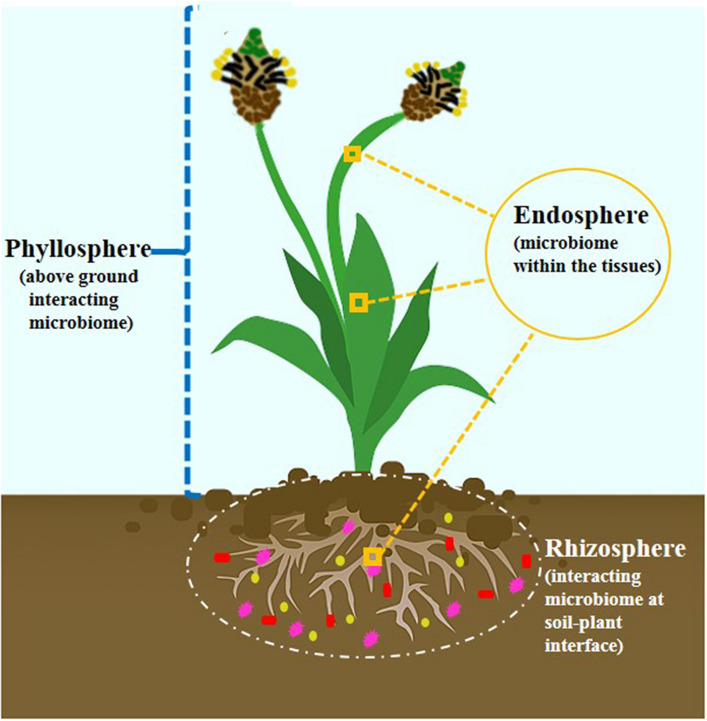
The three main microecosystem involved in plant-microbe interactions.

Decades of research have predicted that plants are naturally colonized by a congregation of microbes. The recognition was converted from single entities to a community conception. These microbes are aggregated in micropockets thriving either outside (rhizosphere), inside (endosphere), or on (phyllosphere) the plant. These three main microecosystems (Figure 1) are regions of chemical communications and are interconnected (Vandenkoornhuyse et al., 2015). The critical interface where plant roots interact intimately with the physicochemical and biotic components of the soil thereby harboring tremendous diversity of microbial communities is referred to as rhizosphere (Wang et al., 2020). Plant-root exudation and microbial substrate utilization traits are the main driving forces for promoting the abundance and metabolic synchronization of microbial communities (Brunel et al., 2020). Santoyo et al. (2016) have reviewed rhizosphere as a nutrient-rich reservoir for facilitating establishment and development of endophytic community. The plant endosphere is defined by complex microbial communities inhabiting and enduring within plant tissues with nonharming nature. Unlike rhizosphere, it receives additional advantage as one habitat without being affected by microenvironment variation and dynamics (Compant et al., 2019). In addition, phyllosphere is another microhabitat which comprises aboveground portions of plant forming an oligotrophic environment for hosting diverse microbial communities.

Evolutionary experiments have underpinned the crucial role of microbial phylotypes in ecosystem functions (Liu et al., 2020). These dynamic microenvironments represent a fundamental way to connect bottom-up and top-down microbial diversity with each other. Collective microbial environments pose additional advantage by coupling Green Revolution and agricultural sustainability. The beneficial microbes in the microhabitat offer multifarious prospects to agroecological systems viz. nutrient solubilization, phytohormone production, nitrogen fixation, bioremediation, improved tolerance to biotic and abiotic stresses, alter plant phenology, modify morphological and size-related traits, and ultimately play a major role in mediating plant responses to climate change and variation (Han et al., 2016; Carrion et al., 2019; Liang et al., 2019). Molecular signaling and trophic interactions between the plant-shaped microhabitat mold the composition and activities of associated microbial populations (Chen X. et al., 2020). Furthermore, mounting evidence have highlighted the potential of beneficial microbes in enhancing plant beneficial traits (Table 1).

**TABLE 1:** Beneficial traits conferred by microbial communities on host plant.

S.No	Microbiome component	Plant	Beneficial traits	References
1.	Rhizosphere	*Phragmites karka*	Lindane (pesticide) degradation, IAA production, ammonia production, and ACC-deaminase activity	Singh and Singh, 2019
		*Brassica nigra*	Antifungal activity against phytopathogens	Kaur et al., 2019
		*Glycine max*	Siderophore, indole acetic acid (IAA), catalase, oxidase, chitinase production, phosphate solubilization, and biocontrol	Prajakta et al., 2019
		*Ipomoea batatas*	Nitrogen fixation and phosphate solubilization	Marques et al., 2019
2.	Endosphere	*Seidlitzia rosmarinus Ehrenb. ex Boiss.*	Salt stress tolerance	Shurigin et al., 2020
		**Anadenanthera colubrina**	Plant growth-promoting traits	Alibrandi et al., 2018
		*Berberis aristata*	Biocontrol potential	Sharma et al., 2018
		**Tamarix chinensis**	Phytoremediation of heavy metals	Chen J. et al., 2020
		**Cymbidium aloifolium**	Phytohormone production	Jagannath et al., 2019
3.	Phyllosphere	*Pyrus serotina, Vitis vinifera, Prunus armeniaca* and *Prunus avium*	Nitrogen fixation	Liang et al., 2019
		*Zea mays*	N2 fixation, phosphate solubilization, production of indole-3-acetic acid, and siderophore	Abadi et al., 2020
		*Oryza sativa*	Drought alleviating potential	Arun et al., 2020
		*Olea europaea*	Antagonistic activity against *Pseudomonas savastanoi* pv. *savastanoi* (Pss)	Mina et al., 2020

Knowledge of the chemical communication between plant and plant, microbe and microbe, and plant and microbe is essential to unveil the complexity of these interactions. Reductionist approaches reveal that plants produce an impressive cocktail of insoluble and soluble low molecular weight organic compounds, known as root exudates/semiochemicals (Quiza et al., 2015; Zhang et al., 2015). This arsenal of secondary metabolites causes inducible structural and physiological modifications in the rhizosphere for the establishment of the microbial communities (Senthil-Kumar and Mysore, 2013). Most of these plant metabolites are synthesized through complex isoprenoid, phenylpropanoid, alkaloid, or fatty acid/polyketide biosynthetic trackways. Flavonoids, coumarins (phytoalexins), benzoxazinoids, strigolactones, terpenoids, malic acid, camalexin, and ethylene are important chemical mediators in the beneficial plant-microbe interaction (Stringlis et al., 2019). The plant exudates are either actively produced or elicited when exposed to certain threats and environmental conditions (Morel and Castro-Sowinski, 2013). These rhizodeposits generate quorum sensing response in microbial communities and also secrete various signaling compounds such as antibiotics, volatile molecules, phytohormones, organic acids, amino acids, sugars, and surface receptors (pattern-recognition receptors) (Zhang et al., 2015; Arif et al., 2020).

Literature reports have addressed the importance of exudates in aboveground and belowground “signalomics,” encouraging symbiosis, minimizing diseases, combating herbivores and pathogen attack, and upregulating growth-promoting traits (Velmourougane et al., 2017; Chagas et al., 2018). The importance of root exudates in modifying the structure and activity of rhizosphere microbiome is well investigated by Lombardi et al. (2018). Their study demonstrated that the roots of stressed plants release peroxidases and oxylipins which act as a chemoattractant for establishing the growth of biocontrol fungus *Trichoderma harzianum*. Shaposhnikov et al. (2020) also demonstrated the role of root exudates in creating a favorable environment for promoting the growth of beneficial rhizobacteria over phytopathogens. They assessed that barley root releases a repertoire of aromatic carboxylic acids as antimicrobial compounds for suppressing the growth of phytopathogenic fungus *Fusarium culmorum.* In contrast, fewer antimicrobial root exudates were released in the presence of antagonistic bacterium *Pseudomonas fluorescens*, therefore, enhancing the assembly of root-beneficial rhizobacteria. Another study conducted by Jin et al. (2019) reported the potential of maize root exudates in inducing chemotactic response, swarming motility, and biofilm formation. Their study establishes that the various components of maize root exudates such as organic acids, amino acids, and sugars significantly enhanced the chemotaxis, cluster movement, and biofilm formation of *Bacillus velezensis* in rhizospheric soil and root tissue. These observations gained significant focus in understanding plant-microbe interaction *via* plant root exudates/semiochemicals. It is worthwhile to understand and manipulate the functions of this “second genome” for future diagnostics and therapy in plants.

## Microbiome Engineering

Amidst climate change and human population growth, the global demand for increasing crop production is expected (Hamilton et al., 2016). For sustainable agriculture, plant-microbial mutualism will help by imparting resistance against biotic and abiotic stresses by enhancing plant health and productivity.

The diversity and composition of microbiota culminates in imparting unique characteristics to the host plant and exert a selection pressure in both natural and managed environments. Lumibao et al. (2020) reported elementary insights on how rhizospheric microbial variation in an engineered ecosystem can greatly influence plant provenance. Their study established the crucial role of feedback mechanisms at plant-soil-microbe interface in shaping the plant intraspecific variation in a depauperate ecosystem. However, the reverse hypothesis recognizing the role of plant genotype in leaving phylogenetic signatures on the rhizosphere microbiomes has been proved experimentally for the first time by Perez-Izquierdo et al. (2019). Their finding establishes a good communication between host-genotypic effects and the environmental effects leading to better performance.

Similarly, engineering the banana endospheric bacterial cell walls (*Enterobacteriaceae*) with 1-aminocyclopropane-1-carboxylate (ACC) deaminase serves as a promising method in imparting resistance against *Fusarium* wilt progression in banana. Synthetic microbial community has been implicated in studying the priority effects and keystone species in the phyllosphere of gnotobiotic *Arabidopsis* model system (Carlstrom et al., 2019). Such a valuable approach helps in testing the fundamental principles responsible for shaping community structure in the phyllosphere. Therefore, engineering the incredible diversity of microbes associated with the host plant serves as a stepping stone in the elucidation of microbiota structure and gene functions. Herein, undermentioned techniques have been enlisted to modify/engineer beneficial microbiome function and services that ensure plant productivity through several generations.

### Soil Amendments

The interconnection of climatic, edaphic, and biotic conditions collectively regulate the physicochemical properties of the soil. Soil structure, organic matter, pH, texture, temperature, quality, and quantity of carbon inputs (root exudates) and nutrient status create conducive environment for the distribution and functional activities of microbial species (Gomez-Sagasti et al., 2018 and Igiehon and Babalola, 2018). Plant metabolites in the root region exert feedback effects on the selection of the microorganisms for colonization. This elicits several responses leading to healthier ecosystem functionality. Such characteristics pay dividend to both plant health and soil-type–specific microbial assembly (Luo et al., 2017).

Soil amendments can include organic additions which may optimize productivity leading to stable agroecosystem function. Organic amendments initiate a cascade of events between different ecosystem trophic levels by manipulating soil biophysical properties, heterogenous distribution of microbial species, and plant traits. The application of organic amendments influences the composition, distribution, and structure of microbial community (Cesarano et al., 2017). They possess multitude advantages propagating with significant changes in soil fertility, plant and microbial biomass, root traits, tissue elemental composition, substrate utilization, and decomposability (Ganesh et al., 2015).

The essential role of organically managed soil systems over conventional systems have been potentially studied by Lupatini et al. (2017). They assessed the composition and diversification of microbial taxa by employing next-generation sequence techniques based on the 16S rRNA gene. Their study provides a comprehensive insight into the phylogenetic richness, diversity, and heterogeneity of soil microbiota being effected by various soil health treatments. This notion that organic amendments positively influence the soil chemistry, microbiota composition, and crop productivity (*Eruca sativa*) was further supported by the study of Bonanomi et al. (2020). Frequent addition of organic substrates like biochar, alfalfa hay, and glucose offers a reliable and effective approach for the sustainable management of soil fertility. Similarly, Obermeier et al. (2020) also studied the effects of pelletized spent mushroom substrate (organic substrate) in combination with mineral fertilizer on agricultural soil health and performance of *Hordeum vulgare* L. Their results highlighted the effect of organic additions in enhancing plant performance and soil bacterial diversity.

Bonanomi et al. (2020) focused on the conducive effects of organic manipulation strategies in inducing plant growth and suppression of plant pathogens. The substantial organic additions work by enhancing the decomposition process which triggers symbiotic microbial colonization and indirectly results into pathogen suppression either by direct parasitism or by production of secondary metabolites with biocontrol potential. The idea of using microbial amendments to enhance crop productivity as investigated by their studies reap potential benefits to the agroecosystem by alleviating the toxic effects of oligotrophication and reduced external inputs. A comprehensive understanding on marked functional implications of soil amendments in manipulating plant-microbe links has been overviewed by Tosi et al. (2020). They reviewed latest trends in optimizing the unique scenario of interactive microbiome at plant-soil interface. Besides organic additions, agricultural practices like rotation, cover crops, intercropping, tillage practices, and multicropping leverage benefit in shaping the diversification of rhizospheric and endospheric microbiota.

In general, application of organic amendments regulates soil nitrogen cycle, improves soil water retention, and improves carbon sequestration and mitigation of climate change effects which ultimately influences plant health and productivity.

### Microbe-Based Manipulations

Host-associated microbial community forms synergistic relationships and displays population-based behavior having immense implications for human food security, biodiversity, and agricultural productivity. These interactions influence the physiology of the host by addressing urgent environmental challenges. Hence, it represents an appealing target for *in situ* microbial engineering.

The frequent application of microbial communities as bioinoculants for increased nutrient mobilization, stress resilience, and plant growth-promoting traits has been well documented (ALKahtani et al., 2020; Alok et al., 2020). Magallon-Servin et al. (2020) designed a consortia of phosphate rock-solubilizing bacteria for testing their capacity in phosphate solubilization, biofilm formation, and root colonization. Their study underscores the promotion of maize seedling growth in low-phosphorus soil amendments under greenhouse conditions by using selected biocompatible multispecies consortia. Similarly, Veeramachaneni and Ramachandrudu (2020) conducted a study for assessing the influence of bioinoculants on oil palm seedlings (*Elaeis guineensis* Jacq.). They reported the efficacy of microbial-based crop amendment in modulating the dynamics and enzymatic potential of rhizospheric microbes thereby promoting the establishment of healthy oil seed crop. Integration of reductionist approaches by vertical transmission of microbial species is of paramount importance in agricultural settings for upregulating plant growth and development, some of the examples of which have been tabulated (Table 2). This is often associated with navigating the plant system for manipulating genetic, biochemical, physical, and metabolic parameters of the plant (Agler et al., 2016; Qiu et al., 2019; Vannier et al., 2019; Khan et al., 2020).

**TABLE 2:** Microbial inoculations enhancing plant growth-promoting traits.

Plant	Beneficial microbe	Plant growth-promoting trait	References
**Bioinoculants/biofertilizers**			
*Lens culinaris var. PL-*	*Chryseobacterium* sp. *PSR10*	Phosphate solubilization	Singh et al., 2018
*Sarracenia* species	Endophytic diazotrophic bacteria	Nitrogen fixation	Sexton et al., 2019
*Zea mays*	Bacterial endophytes (specifically *Bacillus* and *Brevibacillus*)	Enhanced plant growth promotion abilities	ALKahtani et al., 2020
**Stress resilience**			
*Black pepper*	*Bacillus subtilis*, *Bacillus siamensis*, *Brevibacillus gelatini*, *Pseudomonas geniculata*, *Pseudomonas beteli*, *Burkholderia ubonensis*, and *Burkholderia territorii*	Antagonistic to soil-borne *Fusarium solani*	Lau et al., 2020
*Cajanus cajan* and *Festuca*	*Azotobacter chroococcum*, *Bacillus megaterium*, and *Pseudomonas fluorescens*	Impaired growth of *Listeria monocytogenes*	Sharma et al., 2020
*Phaseolus vulgaris*	*Aneurinibacillus aneurinilyticus* and *Paenibacillus* sp.	1-Aminocyclopropane-1-carboxylic acid (ACC) deaminase activity alleviating salinity stress	Gupta and Pandey, 2020
*Oryza sativa*	*Bacillus pumilus* strain JPVS11	Salt tolerance	Kumar et al., 2020
*Avicennia marina*	*Halomonas* sp.	Heavy metal stress	Mukherjee et al., 2019

Recently, there is a surge of interest in accelerating the progress of microbiome research with profound effects on environmental fluctuations. In this context, a series of reductive experiments were carried out for evaluating the capability of endophytic Salicaceae consortia in mitigating abiotic stresses (Aghai et al., 2019). The results of the study affirmed the employment of multispecies consortia as seed or field amendment in garnering a positive conifer seedling performance. Similar to this, Syed et al. (2020) also developed a consortium of plant growth-promoting rhizobacteria (PGPR) in introducing resistance mechanisms against early-stage diseases in *Arachis hypogaea.* Therefore, such an addition of PGPR consortia is not only significant as biocontrol agent but also imparts multitude growth characteristics to the plant.

For addressing the knowledge gaps between microbe and microbe interaction dynamics, synthetic biology has emerged as an effective probe. It is a field for designing, predicting, and manipulating the behavior of natural microbial populations with focus on specific applications. Recent advancements in next-generation sequencing (NGS) platform, gene editing technologies, culture independent analysis, meta-“omics” and bioinformatics tools have provided a top–down approach for creatively synthesizing synthetic microbial consortia. Synthetic biologists are honing their ability for revolutionizing agricultural productivity by introducing genes from other photosynthetic microorganisms or by *in vivo* construction of synthetic metabolic pathways (Ogawa et al., 2015; Long et al., 2016; Schwander et al., 2016).

Recently, a novel strategy was introduced known as synthetic microbial communities (SynComs) based on tailoring plant-microbe interactions by recruiting and manipulating microbial consortia with enhanced colonization, prevalence, and stress-resilient traits. Such synthetically designed microbial inoculants bridge ecology and genetics of microbial communities in sustainable agricultural outcomes (de Souza et al., 2020). Such microbial-based manipulations hold great promise in challenging the global stagnation in terms of crop production, biomass requirement, and climate uncertainties.

### Plant-Based Manipulations

To address the global and environmental challenges including climatic variations, desertification, salinization, emergence of new diseases, and resistance to biotic and abiotic stresses, there is a need to look for holistic and sustainable approaches that ensure the global conservation of food resources (Garcia et al., 2020 and Ulian et al., 2020). In this context, plant-based manipulation strategies focused on advanced molecular biology techniques, biochemical approaches, mutagenesis, selection, and breeding of crop varieties have significantly unraveled the plant system against various environmental changes (Table 3). Furthermore, the continued success of renowned Green Revolution can be achieved by high-throughput transcription profiling, identification of specific protein network on large scale, molecular modeling and their association with environmental changes for improved agricultural traits and increased yields (Mahfouz et al., 2016; Jangra et al., 2017).

**TABLE 3:** Methods adopted for plant based microbiome engineering.

S.No.	Method	Strategy	References
1.	Synthetic selection	Designing a microbiome comprising of few culturable microbial strains serving as a streamline model for unraveling the fundamental principles of plant-microbe interaction	Vorholt et al., 2017
2.	Artificial microbiome selection	Synthetically selecting multispecies assemblages (culturable and nonculturable) with desirable traits for optimizing functional dynamics of microbial communities	Wright et al., 2019
3.	Single-cell genomics, whole-community metagenomics, and metaproteomics	Orchestrating genomic data with transcriptomic and metabolomic data for improving individual microbiome function and network interactions.	Hadadi et al., 2020
4.	Metabolic network monitoring	Designing models at species level for predicting the dynamic shifts in microbe-microbe interaction in response to substrate gradients, metabolic dependence, competition, and spatial heterogeneity	Kessell et al., 2020

With the emergence of site-specific nucleases (TALENs), targeted mutagenesis, genome-editing tools like CRISPR/Cas systems, the discovery of novel traits, trait development, site-specific genome modifications have been enabled across the plant kingdom. The use of CRISPR/Cas systems in the targeted engineering of crop traits has been well studied. Butt et al. (2018) studied the CRISPR/Cas9-mediated targeted disruption of *carotenoid cleavage dioxygenase* (ccd7) in rice (*Oryza sativa*), controlling a key step in strigolactone biosynthesis and resulting in increased tillering ccd7 mutants. Such an unprecedented engineering of plant architecture traits underpins the single-base level control of CRISPR/Cas systems. In concomitant with the previous study, IR64 rice lines resistant to rice tungro spherical virus (RTSV) were developed by targeted mutagenesis of *eIF4G*. The CRISPR/Cas9-induced mutations resulted in the generation of novel *eIF4G* alleles for the production of RTSV resistance varieties (Macovei et al., 2018). The application of CRISPR/Cas9 system has also been reported in preferable endogenous gene modifications (Okuzaki et al., 2018). Gene editing of an agronomically targeted *fatty acid desaturase 2* gene (*FAD2*) in *Brassica napus* using the CRISPR/Cas9 system resulted in desirable mutant alleles with increased oleic content. Such a cutting edge plant-genome engineering techniques stand poised in shaping and sustainably conserving the architecture of “Green Revolution” (Sedeek et al., 2019).

Simkin et al. (2017) generated transgenic *Arabidopsis* plants with altered combinations of sedoheptulose 1,7−bisphosphatase (SBPase), fructose 1,6−bisphophate aldolase (FBPA), and the glycine decarboxylase−H protein (GDC−H) gene coding for three different enzymes of the Calvin–Benson cycle and photorespiratory pathway. This multigene stacking results in cumulative increase in photosynthesis, biomass, and seed yield. Designing synthetic carbon-conserving photorespiration bypass routes by metabolic engineering can also be used as a strategy in this direction. Trudeau et al. (2018) used this strategy for two enzymes namely, acetyl-CoA synthetase and propionyl-CoA reductase. These enzymes enable the formation of glycolate reduction module, i.e., recycling of glycolate to ribulose 1,5-bisphosphate without the loss of CO_2_. Lopez-Calcagno et al. (2019) engineered tobacco plants for increased biomass by overexpressing photorespiration-limiting H-protein of the glycine cleavage system using the leaf−specific promoter ST−LS1. CRISPR/Cas9−mediated editing of SlJAZ2 has been used to prevent stomatal reopening and thus providing resistance. Ortigosa et al. (2019), designed tomato resistant varieties against *Pseudomonas syringae* pv. tomato (Pto) DC3000 pathogen (causing bacterial speck disease) using this strategy.

The prerequisite for targeted improvement of traits is the use of molecular scissors capable of introducing site-specific double-stranded breaks (DSBs). Such nuclease-induced breaks stimulate the cell’s DNA repair machinery for repairing these DSBs either by nonhomologous end-joining (NHEJ) or by homology-directed repair (HDR). NHEJ results in frameshift mutations creating functional knockouts of a gene. Unlike NHEJ, HDR introduces specific point mutations or a new gene sequence by precisely modifying/repairing genome with oligonucleotide template with homologous ends or arms to the DSB flanking sequence. Such programmable site-specific techniques are used for targeted improvement of crop traits (Butt et al., 2018; Sedeek et al., 2019). Therefore, harnessing engineered variants of eukaryotic genes will pave way for significant improvement in plant yield and food security. Also, key advances in functional genomics will anticipate forward synergies in genomics and plant-breeding techniques for improved crop genetic resources, new cultivar development, and targeted crop improvement.

From an agronomic perspective, key gene players of biosynthetic and metabolic pathway can be targeted at the transcriptional, posttranscriptional, and posttranslational levels for the manipulation and better understanding of pathways. Gene clusters like *Phenylalanine ammonia lyase* (*PALs*), *Flowering Locus T-Like* (*FTL*), *Calcium–dependent protein kinases* (*CDKs*), and other regulatory networks has undergone substantial editing with better performance-tailored plants (Nagegowda and Gupta, 2020; Yadav et al., 2020 and Schulz et al., 2021).

Furthermore, the introduction of microbial genes in plant system for improving biomass yield and photosynthetic efficiency has been well presented in the study of Hay et al. (2017). They transformed the soybean (*Glycine max* cv. Thorne) with cyanobacterial inorganic carbon transporter B (*ictB*) gene and found that transgenic plant showed increased photosynthetic rates and biomass production. In accordance with this finding, constitutive expression of cyanobacterial fructose-1,6/sedoheptulose-1,7-bisphosphatase in *Glycine max* leads to significantly higher carbon assimilation and seed yield under elevated CO_2_ and temperature conditions as reported by Kohler et al. (2017). Not only this, diverse strategies focusing on improving nutrient uptake, use of broad-host range plasmids for engineering microbes, and improved CO_2_ fixation efficiency were also targeted. The genetic transformation of cytochrome c6 (UfCyt c6) gene from *Ulva fasciata* Delile in tobacco was reported for the first time by Yadav et al. (2018). The study reported higher quantum yields of PSII, stomatal conductance, and transpiration rate in transgenic tobacco plants, hence, providing deeper insights into *in vivo* realization of photosynthetic enhancement by improving photosynthetic electron transfer and water use efficiency. Another feasible approach focused on improving carbon fixation efficiency was studied by Yu et al. (2018). They reported the *in vitro* designing and investigated the *in vivo* functional demonstration of synthetic malyl-CoA-glycerate (MCG) pathway in *Escherichia coli* and photosynthetic organism *Synechococcus elongates* PCC7942o for optimal synthesis of acetyl-CoA. Nonetheless, the elucidation of data from omics−based system biology has come up as an innovative and powerful tool for heterologous engineering of plant traits.

Therefore, deep understanding of various techniques at soil, microbe, and plant level will surely open a new window in harmonizing the complex assemblages of phytomicrobiome. Furthermore, combined analysis will act as selective force in sculpting intrinsically complex, heterogenous, and dynamic microbiome assembly. However, these manipulation strategies are hindered by current technical gaps, thereby hampering future commercialization and adoption strategies. These include root exudate composition, root architecture, induced systemic response, competition by indigenous soil microflora (at root-soil interface), poor microbial colonization, environmental stress conditions, limited persistence, inability of mutual recognition (at plant-root interface), host genotype, lack of *in situ* manipulation tools and standardization protocols (at the host level). These knowledge gaps can further be addressed by QTL mapping and integration of various meta-omics approaches (metagenomics, metatranscriptomics, and metabolomics). Implementation of synthetic biology techniques for the discovery of novel phages, plasmids, and transposable elements for *in situ* microbiome engineering will possibly help in the establishment and success of healthier microbiomes.

## Concluding Remarks

In view of the complexities of microbial interactions, “microbe-friendly” plants or genetically engineered/edited plant genome will lead to field success by strengthening plant health and preparedness against environmental fluctuations. This microbial fortification will enhance gene expression, enzymatic parameters, nutrient uptake, and biocontrol response. Thus, strategic manipulation and inoculation of microbial multispecies will have more impactful response on plant growth performance by minimizing chemical farm inputs. In this scenario, microbiome engineering offers exciting opportunities for understanding and engineering individual organisms to the entire ecosystems. This envisioned technology will reveal a vast diversity and elegance underlying natural microbial ecosystems at the frontier of nutritional and ecological demands. The transcendental role of microbes in the field of basic sciences, human health, and agriculture is worth mentioning at the frontier of synthetic biology strategies. However, many of the associated technologies of bioengineering are still in its infancy and require a regulated framework for examining its future in crop improvement programs.

## Author Contributions

All authors listed have made substantial, direct and intellectual contribution to the work, and approved it for publication.

## Conflict of Interest

The authors declare that the research was conducted in the absence of any commercial or financial relationships that could be construed as a potential conflict of interest.
